# 3D fascicular reconstruction of median and ulnar nerve: initial experience and comparison between high-resolution ultrasound and MR microscopy

**DOI:** 10.1186/s41747-024-00495-5

**Published:** 2024-08-28

**Authors:** Luka Pušnik, Lisa Lechner, Igor Serša, Erika Cvetko, Philipp Haas, Suren Armeni Jengojan, Žiga Snoj

**Affiliations:** 1 Institute of Anatomy, Faculty of Medicine, University of Ljubljana, Ljubljana, Slovenia; 2 Division of Neuroradiology and Musculoskeletal Radiology, Department of Biomedical Imaging and Image-Guided Therapy, Medical University of Vienna, Vienna, Austria; 3 Department of Condensed Matter Physics, Jožef Stefan Institute, Ljubljana, Slovenia; 4 Department of Radiology, Faculty of Medicine, University of Ljubljana, Ljubljana, Slovenia; 5 Institute of Radiology, University Medical Centre Ljubljana, Ljubljana, Slovenia

**Keywords:** Artificial intelligence, Imaging (three-dimensional), Magnetic resonance imaging, Peripheral nerves, Ultrasound

## Abstract

**Background:**

The complex anatomy of peripheral nerves has been traditionally investigated through histological microsections, with inherent limitations. We aimed to compare three-dimensional (3D) reconstructions of median and ulnar nerves acquired with tomographic high-resolution ultrasound (HRUS) and magnetic resonance microscopy (MRM) and assess their capacity to depict intraneural anatomy.

**Methods:**

Three fresh-frozen human upper extremity specimens were prepared for HRUS imaging by submersion in a water medium. The median and ulnar nerves were pierced with sutures to improve orientation during imaging. Peripheral nerve 3D HRUS scanning was performed on the mid-upper arm using a broadband linear probe (10–22 MHz) equipped with a tomographic 3D HRUS system. Following excision, nerves were cut into 16-mm segments and loaded into the MRM probe of a 9.4-T system (scanning time 27 h). Fascicle and nerve counting was performed to estimate the nerve volume, fascicle volume, fascicle count, and number of interfascicular connections. HRUS reconstructions employed artificial intelligence-based algorithms, while MRM reconstructions were generated using an open-source imaging software 3D slicer.

**Results:**

Compared to MRM, 3D HRUS underestimated nerve volume by up to 22% and volume of all fascicles by up to 11%. Additionally, 3D HRUS depicted 6–60% fewer fascicles compared to MRM and visualized approximately half as many interfascicular connections.

**Conclusion:**

MRM demonstrated a more detailed fascicular depiction compared to 3D HRUS, with a greater capacity for visualizing smaller fascicles. While 3D HRUS reconstructions can offer supplementary data in peripheral nerve assessment, their limitations in depicting interfascicular connections and small fascicles within clusters necessitate cautious interpretation.

**Clinical relevance statement:**

Although 3D HRUS reconstructions can offer supplementary data in peripheral nerve assessment, even in intraoperative settings, their limitations in depicting interfascicular branches and small fascicles within clusters require cautious interpretation.

**Key Points:**

- 3D HRUS was limited in visualizing nerve interfascicular connections.
- MRM demonstrated better nerve fascicle depiction than 3D HRUS.
- MRM depicted more nerve interfascicular connections than 3D HRUS.

**Graphical Abstract:**

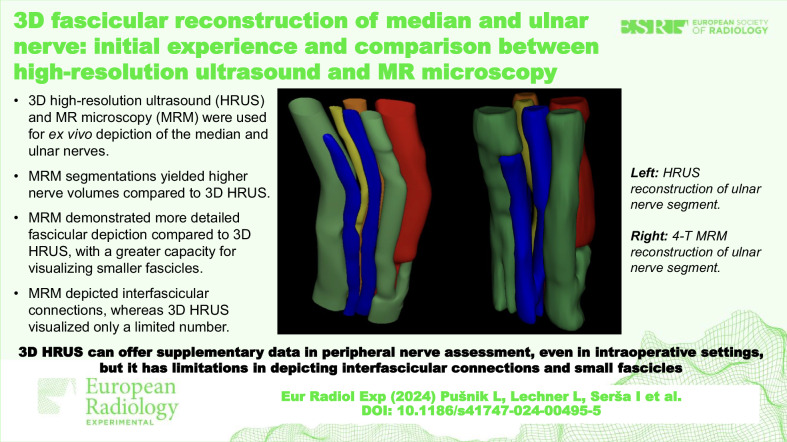

## Background

High-resolution ultrasound (HRUS) enables noninvasive, real-time visualization of peripheral nerves, making it a valuable adjunct in diagnosing and treating peripheral nerve disorders. Its wide availability, cost-effectiveness, and excellent spatial resolution have solidified its utility in peripheral nerve imaging [1–3], providing valuable morphological and structural information to supplement electrophysiologic findings [4].

The complex fascicular architecture of peripheral nerves has traditionally been investigated through microdissection, which has enabled the description of complex fascicular branching [5]. Nevertheless, accurately depicting such structural dynamics in two-dimensional (2D) imaging can be challenging and necessitates modalities that enable three-dimensional (3D) visualisation. Various registration techniques have been developed for the 3D rendering of histological samples; however, such methods generally require extensive pre- and post-processing [6, 7]. Additionally, histological samples can undergo structural alterations due to excision, freezing-thawing, fixative shrinkage, and cryostat manipulation, necessitating the exploration of alternative techniques to improve the depiction of intraneural anatomy [8–11]. Magnetic resonance microscopy (MRM) is one such technique that has demonstrated the accurate depiction of more than 90% of median and ulnar nerve fascicles compared to histological cross-sections [12]. This method does not require extensive sample processing or manipulation and is superior to HRUS in fascicular depiction [9].

While previous 3D ultrasonography (US) studies focused on morphometric values such as the cross-sectional area (CSA) [13], none have directly compared these findings to other modalities or fully explored the potential of 3D US for depicting intraneural anatomy. 3D US has emerged as a potential tool to reduce operator dependency, partially addressing limitations of traditional B-mode US, such as vulnerability to accidental probe tilt [13–16]. Acquisition with 3D US is rapid and provides high spatial resolution, allowing for flexible reconstruction of the segment with surrounding tissue in any plane [17]. Studies have demonstrated the ability to depict the median, ulnar, and radial nerve in the forearm or wrist using the 3D US [13, 17, 18]. Nevertheless, further exploration of 3D HRUS capabilities is crucial to fully understand the limits of *in vivo* intraneural anatomy depiction.

The majority of closed traumatic nerve injuries involve lesions in continuity rather than complete nerve transection [19]. Consequently, intraoperative fascicle visualization can aid in detecting fascicular involvement, influencing clinical decisions, and potentially determining whether traumatic lesions necessitate surgical intervention [20]. In nerve surgeries, surgical loupes are often utilized for less complex procedures, offering magnifications between 2.5 × and 6 ×, with a resolution of approximately 100 µm. For more complex cases, operating microscopes are indispensable, providing magnifications from 6 × to 40 ×. Conversely, HRUS can also achieve a resolution of about 100 µm, sufficient to identify individual nerve fascicles [21, 22]. While 3D tomographic US shows promise for intraoperative nerve and fascicle visualization, thorough validation against other modalities is essential to ensure reliability. Accordingly, this study aimed to compare 3D US reconstructions of peripheral nerves with high-field MRM reconstructions and determine the capacity of 3D US in depicting intraneural anatomy.

## Methods

### Ethical approval

The upper extremity specimens were obtained from three fresh frozen anatomical cadavers donated to the Institute of Anatomy, Faculty of Medicine, University of Ljubljana, Slovenia, through a willed cadaver donation programme. A written informed consent was obtained before death from a person donating themselves *post-mortem* for research purposes. None of the donors had any known peripheral nerve disorder. The study was approved by the Republic of Slovenia National Medical Ethics Committee (approval no.: 0120-239/2020/3).

### Sample preparation

**Fig. 1:**
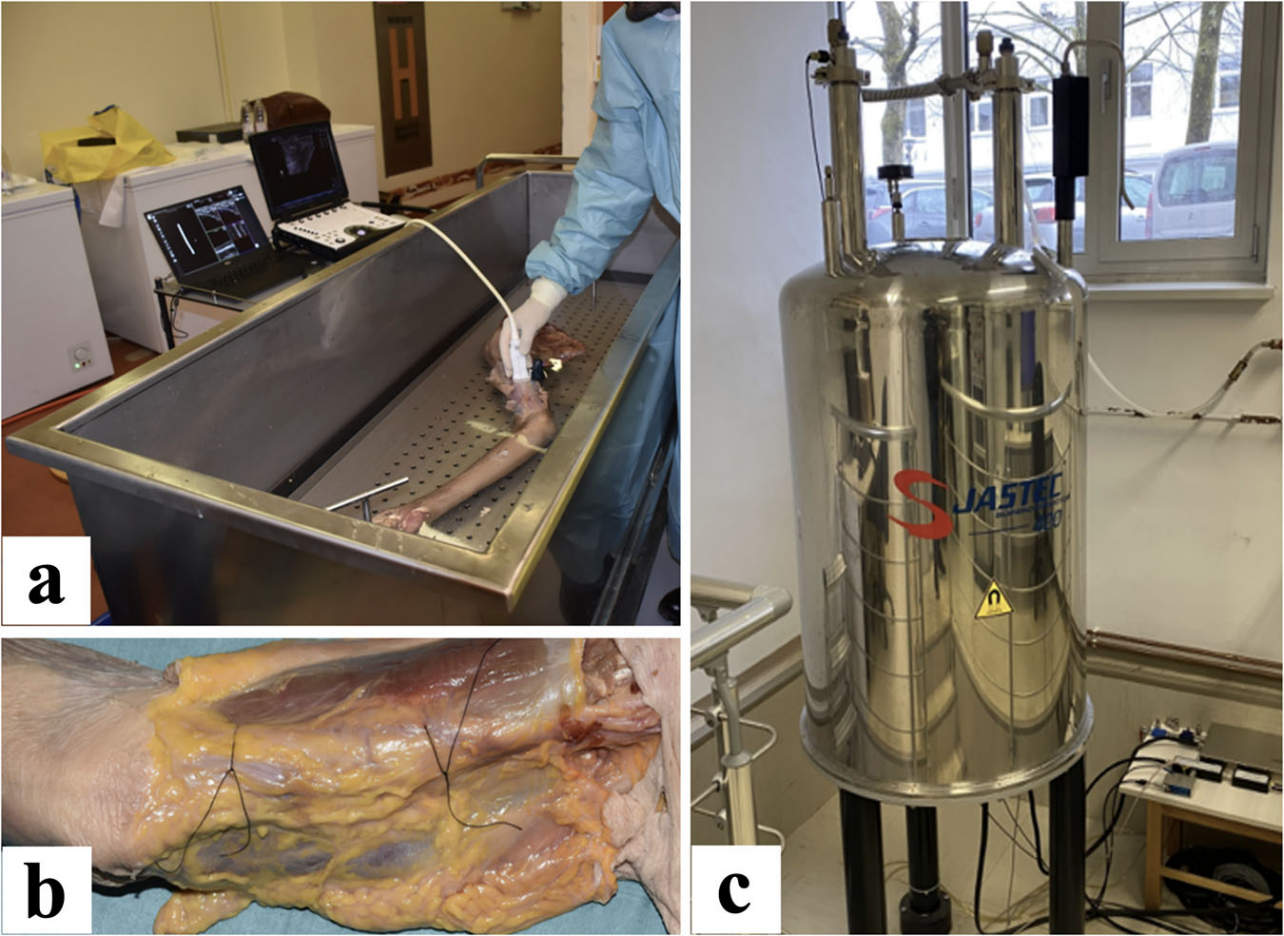
HRUS and MRM of the upper extremity nerves. **a** Depicts a tomographic HRUS setting with the upper extremity submerged within the water tank while performing the scan of the median nerve. The probe is set in the mid-upper arm, perpendicular to the nerve axis with minimal pressure applied. **b** The median nerve is pierced within the upper arm and skin with subcutaneous tissue partly removed. The nerve is marked with one proximal and one distal suture that later served for better orientation during the excision and cross-referencing process. **c** Shows a 9.4-T superconducting vertical bore magnet of an MRM system during the scanning process

Three upper limb specimens, including the complete scapula and clavicle, were harvested. Each extremity was put on a metallic tray (dimensions: 140 cm × 45 cm), with the palm rotated upwards, forearm supinated, and elbow joint in partial flexion. The tray was submerged into a water-filled tank (dimensions: 150 cm × 50 cm × 40 cm) with a temperature of 20 °C that served as an optimal US media. A GE Logiq E US system (GE Healthcare, Milwaukee, WI, USA) with broadband linear probe (10–22 MHz) equipped with a tomographic 3D US (PIUR tomographic US Infinity system) was employed for depicting one nerve in each extremity (one ulnar and two median nerves) in the middle third of the upper arm (Fig. 1a). The skin was removed before the acquisition. Each nerve was punctured and marked using two braided sutures made of silk (USP 3/0, GS 60 mm, straight cutting, non-absorbable; SMI AG, St. Vith, Belgium) that served as a marker for nerve excision and cutting, as well as for precise cross-referencing with MRM (Fig. 1b).

### 3D US

After nerve marking, US scanning was performed with the following parameters: A GE L10-22 MHz transducer with a standardized “peripheral nerve” US preset (B-mode, frame rate: 64 frames per second, frequency: 22 MHz, gain: 44, depth: 1 cm, time-gain compensation: centred, dynamic range (compression): 72, auto optimization: 100%). Gain, focus, depth, frequency, and time-gain compensation were then adapted to the individual anatomical situation. The US scanner was equipped with a free-hand tomographic 3D HRUS system that functions by capturing image frames from a standard US system. The probe sensor was attached to the US probe to track its position as the structure of interest was examined. To determine movement direction, the software employs convolutional neural networks in its processing algorithms to accurately calculate velocities and accelerations. These algorithms filter out motion artefacts, ensuring precise distance measurements. The nerves were scanned by a musculoskeletal radiologist (S.A.J.) with ten years of subspecialty expertise in peripheral nerve imaging. An approximately 10-cm long segment between the first and last suture of the nerve was scanned (Fig. 1a). Promptly after the US scan, the segments between the first and last suture were excised, and from each nerve, a 16-mm long sample with either proximal or distal suture was prepared for MRM acquisition.

### MRM acquisition

MRM was performed on a 400-MHz system consisting of a 9.4-T superconducting vertical bore magnet from Jastec (Tokyo, Japan) (Fig. 1c), Tecmag Redstone NMR/ Magnetic resonance imaging (MRI) spectrometer (Houston, TX, USA) and Bruker Micro 2.5 gradient system and RF probes (Ettlingen, Germany). All three samples were simultaneously placed into a 20-mm-diameter tube and inserted into an MRM probe in the magnet. The parts of nerves with sutures were positioned towards the bottom of the glass tube, with plastic inserts preventing bending. To avoid desiccation, the nerves were submerged in a perfluorinated fluid (Galden SV90, Solvay, Brussels, Belgium) [23]. The images were acquired using a 3D short-tau inversion-recovery (STIR) imaging sequence with the following parameters: repetition time/echo time = 2,900 ms/6 ms; scanned volume = 19 × 9.5 × 16 mm^3^; acquisition matrix (*x* × *y* × *z*) = 512 × 256 × 32; inversion time = 415 ms; slice thickness = 500 μm; interslice gap = 0 μm; number of slices = 32; and four number of signal averages. The MRM scanning time was 27 h. STIR sequence provides high contrast between the fascicles and interfascicular epineurium, a characteristic that allows the delineation process and 3D rendering.

### Image analysis

The fascicle and nerve CSAs were delineated on MRM and 2D US images. On MRM, fascicles were considered as oval or round structures circumferentially surrounded by hyperintense rim representing the perineurium, and interfascicular epineurium was considered as connective tissue between the fascicles and hypointense and circularly shaped epineurium [12]. Nerve CSA was tracked with delineation of all fascicles and interfascicular epineurium in each slice; however, without extra connective tissue or background beyond the epineurium. The contouring of MRM slices was performed using the ImageJ software (National Institutes of Health, Bethesda, MD, United States). On 2D US images, the fascicles and nerve CSA were separately delineated on slices, with the number depending on the quality of US scans. The hyperechoic oval and round structures were considered nerve fascicles, while the hypoechoic rim surrounding the fascicles was considered epineurium surrounding the nerve [12].

The HRUS images and 3D volumes were obtained with a tomographic HRUS system with artificial intelligence-based image reconstruction algorithms [24]. The MRM reconstructions were rendered using 3D Slicer (The Slicer Community, Boston, MA, USA) [25]. From 3D HRUS images, the volume for a predefined nerve segment and the volume of all fascicles were estimated by CSA segmentation on individual 2D HRUS images and subsequent automatized 3D reconstruction. The nerve and/or fascicle volume for MRM acquisition was calculated with the mean CSA of all slices multiplied by the thickness of the analyzed segment. On HRUS, the volume for each fascicle in the predefined area of interest was calculated individually using the tomographic US software, with the volumes being summed. For each modality, a fascicle volume ratio (FVR) was calculated as a quotient between the total volume of all fascicles and the volume of the nerve. The number of interfascicular connections was evaluated on 2D images and defined as the number of fascicles approaching another fascicle and exchanging the fibres, which resulted in altered cross-sections of both fascicles in consequent slices.

### Statistical analysis

Data are expressed as means ± standard deviations or proportions (%), and simple analysis has been performed using Microsoft Office Excel (Redmond, WA, USA). The statistical tests for comparing the measurements were not performed due to the explorative nature of the study and limited sample size. To assess inter- and intra-rater reliability, 2D HRUS and MRM slices were re-delineated two months after the initial outlining by the same observer and second independent evaluator. Intra-class correlation coefficient (ICC) for manual delineation of fascicles and nerves was calculated with SPSS (SPSS Inc., Chicago, Illinois, USA) and interpreted according to the guidelines [26, 27].

## Results

**Fig. 2:**
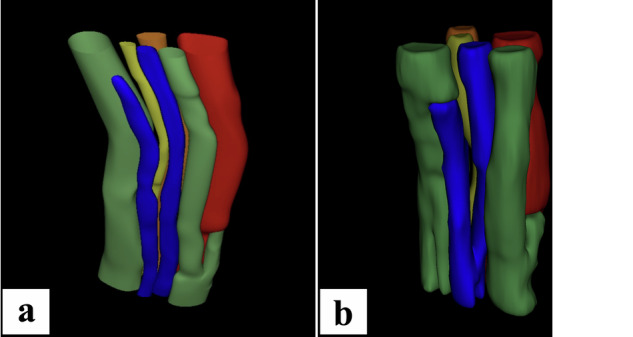
3D fascicular anatomy of the peripheral nerve. **a** Represents a 3D HRUS reconstruction of the ulnar nerve. Eight different fascicles can be noted throughout the segment, and only one interfascicular connection can be seen on the 3D reconstructed image from this angle, namely between the red and green fascicles. The red fascicle can be seen throughout the portrayed segment, partly hidden behind the green fascicle. Note that the fascicle distortion is caused by external factors such as the pressure of the probe. **b** Depicts a MRM image of the same nerve with more interfascicular branches. For instance, the interfascicular connections between the blue fascicles can be seen with one of the fascicles consequently merging with the green fascicle. Conversely, this interfascicular branching of the blue fascicles cannot be noted on HRUS and the left blue fascicle cannot be properly depicted in the upper segment of the reconstructed image. The interfascicular connection of the blue fascicles can be observed on MRM, while it was not depicted on 3D HRUS. The fascicle distortion is caused due to the slightly oblique position of the nerve in the MRM probe

**Fig. 3:**
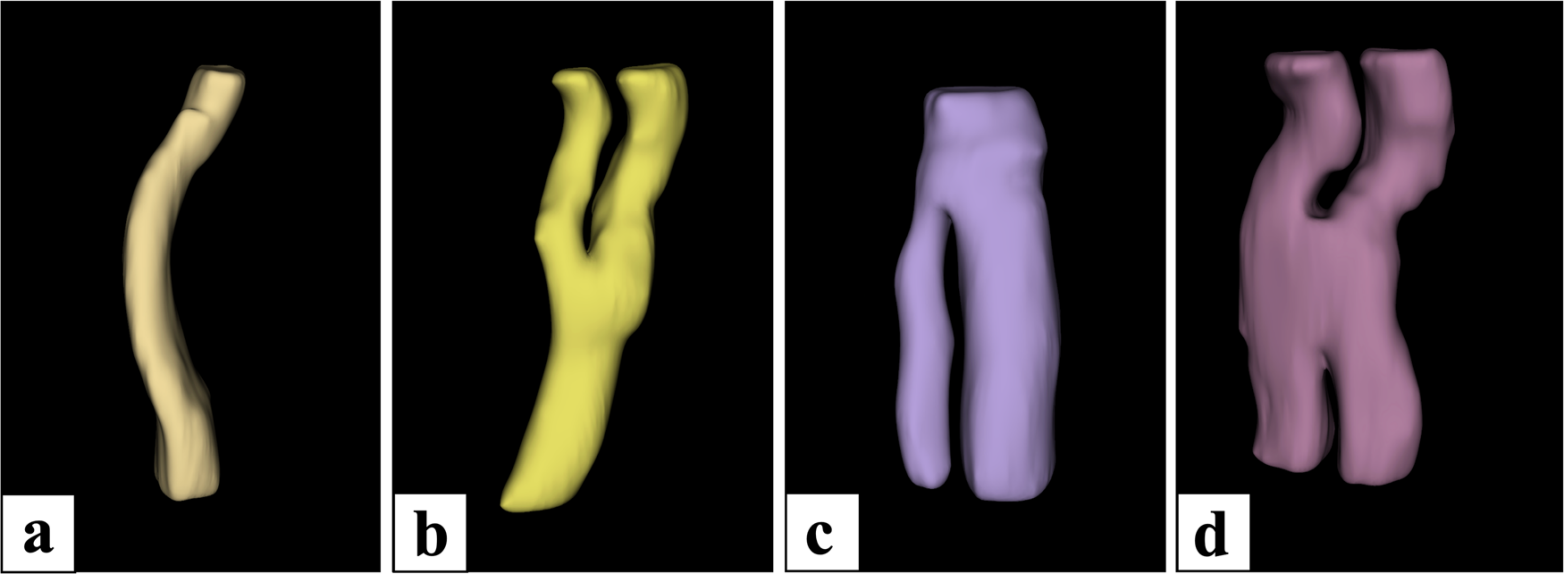
Types of interfascicular connections observed on MRM-rendered images. **a** Depicts an isolated single fascicle, (**b**) a Y-shaped pattern, (**c**) an inverted Y-shaped pattern, and (**d**) an X-shaped or complex type interfascicular connection pattern. Note that the figure depicts the isolated fascicles of shorter lengths than the analyzed nerve segment

The 3D HRUS underestimated nerve volume by up to 22% and volume of all fascicles by up to 11% compared to MRM (Table 1). MRM visualized a higher proportion of smaller nerve fascicles compared to 3D HRUS, which tended to depict them as fascicle clusters. In one median nerve, approximately half of the fascicles were not recognized as single fascicles. The 3D HRUS could only depict some of the interfascicular connections apparent on MRM. The estimated volume of individual nerve and all fascicles combined, number of fascicles, FVR, and number of interfascicular connections are presented in Table 1. Inter- and intra-rater reliability for delineating structures was good to excellent (Table 2). 3D reconstruction images representing the fascicular anatomy of the ulnar nerve are depicted in Fig. 2, while interfascicular connection types observed on MRM images are shown in Fig. 3.

**Table 1:** Estimated nerve volume, volume of all fascicles, number of fascicles, FVR, and interfascicular connections

	Nerve I	Nerve II	Nerve III
	Median nerve	Median nerve	Ulnar nerve
Estimated nerve volume 3D HRUS^1^ [mL]	0.14	0.14	0.09
Estimated nerve volume MRM^1^ [mL]	0.14	0.18	0.11
Δ volume [mL] (%)	0.00 (0%)	0.04 (22%)	0.02 (18%)
Estimated volume of fascicles 3D HRUS^1^ [mL]	0.09	0.08	0.07
Estimated volume of fascicles MRM^1^ [mL]	0.09	0.09	0.07
Δ volume all fascicles [mL] (%)	0.00 (0%)	0.01 (11%)	0.00 (0%)
FVR for 3D HRUS	0.64	0.57	0.78
FVR for MRM	0.64	0.50	0.64
Δ FVR (%)	0.00 (0%)	0.07 (14%)	0.14 (22%)
No. fascicles 3D HRUS^2^	3.64 ± 0.49	5.00 ± 0.00	6.67 ± 0.50
No. fascicles MRM^3^	4.40 ± 0.52	12.50 ± 1.84	7.10 ± 0.74
Δ Fascicles (%)	0.76 (17%)	7.50 (60%)	0.43 (6%)
Number of interfascicular connections at 3D HRUS	5	5	4
Number of interfascicular connections at MRM	11	11	9
Δ Interfascicular connections (%)	6 (54%)	6 (45%)	5 (55%)

*3D HRUS* Three-dimensional high-resolution ultrasound, *FVR* Fascicle volume ratio, *MRM* Magnetic resonance microscopy, *Δ* difference between the modalities

^1^ Estimated volume of nerve/fascicles was calculated for a 16-mm-long segment

^2^ Evaluated on 2D HRUS slices and expressed as a mean value with a standard deviation

^3^ Evaluated on 2D MRM slices and expressed as a mean value with a standard deviation

**Table 2:** Inter- and intra-rater reliability

		Intra-rater ICC^a^	Inter-rater ICC^a^
HRUS	Fascicle count	0.95	0.94
	Fascicular CSA	0.95	0.92
	Nerve CSA	0.89	0.84
MRM	Fascicle count	0.98	0.98
	Fascicular CSA	0.94	0.89
	Nerve CSA	0.99	0.99

*CSA* Cross-sectional area, *HRUS* High-resolution ultrasound, *ICC* Intraclass correlation coefficient, *MRM* Magnetic resonance microscopy

^a^ICC was calculated from ten different cross-sections, separately for MRM and HRUS delineations [26]

## Discussion

This study employed 3D HRUS and MRM for median and ulnar nerve *ex vivo* depiction in the upper extremities. MRM segmentations yielded higher volumes of nerves compared to the 3D HRUS. MRM demonstrated a more detailed fascicular depiction compared to 3D HRUS, with a greater capacity for visualizing smaller fascicles. MRM could also depict interfascicular connections, whereas 3D HRUS visualized only a limited number.

Compared to the MRM, 6–60% of fascicles were not differentiated on the tomographic 3D HRUS images. This wide interval could be attributable to a difference in the intraneural anatomy of the analyzed nerve segments. The first and third nerves had fewer fascicles in comparison to the second nerve, with the difference in fascicular depiction between both imaging modalities being minor. Conversely, the second nerve had the highest number of smaller fascicles, which failed to be depicted due to the lower resolution of 3D HRUS compared to 9.4-T MRM. Prior studies demonstrate that US, even with HRUS, exhibits a restricted capacity for depicting small fascicles [12].

Small fascicles might not be distinguishable from the surrounding interfascicular epineurium, or clusters of fascicles may appear as a single undifferentiated structure on US [12, 28, 29]. Larger interfascicular distances facilitate the fascicular differentiation, presumably due to the amount of interfascicular tissue [12, 30]. This notion is crucial because variations in image acquisition methods must also be considered when interpreting disparities in fascicle depiction. Some degree of probe pressure is needed for US depiction, and thus, a small degree of nerve distortion is expected with some effect on fascicle differentiation. The US acquisition was performed under the water with the probe sliding and requiring minimal pressure to minimize this effect.

The estimated nerve volume of MRM-acquired images was higher compared to the 3D HRUS. This could partly be attributed to the compression and deformation of soft interfascicular epineurium or even the inclusion of the background tissue while delineating the nerves on MRM slices. Fascicle volume differed less than whole-nerve volume. This may be due to the greater resistance of the perineurium to deformation compared to interfascicular epineurium, limiting the change in shape caused by US probe pressure [31]. The more comparable results of fascicular volumes compared to the nerve volumes could also stem from the outer rim of the perineurium being more discernible than the epineurium in the US images [10].

As the volumetric methods, such as the estimated nerve or fascicular volume, depend on the length of the analyzed segment, relative measurements such as FVR should be preferred. Such volumetric measurements could enhance understanding of nerve pathologies, enable better comparison, and can be of special value in hospitals where technicians perform the scan independently, with the clinicians evaluating them later [13, 32]. The volumetric measurement methods might be relevant in evaluating the course of therapy of peripheral nerve pathologies with single or multiple fascicle involvement. Considering the alterations in volume, the tomographic US as a simple and cost-effective alternative imaging modality in clinical or intraoperative settings may contribute to understanding both the spatial distribution and temporal progression of peripheral nerve damage [33]. Specifically, acute injuries may manifest as nerve and fascicular enlargement, indicating axonal disruption, endoneurial, and perineurial oedema. In chronic neuropathies, changes in fascicle volume occur due to fibre loss, alongside the proliferation of endoneurial collagen and thickening of the perineurium. Histopathologic analysis of diabetic neuropathy has already validated these changes, which correlate with prolonged fascicular involution and intrapreneurial fatty and fibrous substitution [34].

The exploration of 3D HRUS for the visualization of nerve fascicles represents a novel and intriguing area of research. Our study highlights the potential of 3D HRUS to deliver more accurate and reliable imaging compared to traditional 2D US. Enhanced visualization of nerve fascicles could profoundly impact clinical and surgical decision-making processes, offering superior diagnostic capabilities and potentially improving patient outcomes. This advancement is particularly relevant for procedures requiring detailed mapping of nerves and nerve fascicles, such as nerve reconstruction, direct nerve restoration, nerve transfer, and autografting [35, 36]. However, it is imperative to acknowledge these results as preliminary. While the initial findings are promising, they are still in the early stages of validating 3D HRUS as a reliable tool for nerve visualization. As the results of this study reflect intraoperative settings, the fascicular depiction ability of 3D HRUS cannot be directly extrapolated to ambulatory settings and should be interpreted with caution. Further research is needed to confirm these results in larger and more diverse sample populations, including *in vivo* patients. Additionally, comprehensive studies comparing 3D HRUS with other advanced imaging modalities, such as MR neurography or 3-T MRI, are essential to establish its relative efficacy and practical advantages. Future research should also focus on standardizing imaging protocols and postprocessing techniques to minimize variability and enhance reproducibility.

This study had some limitations which can be addressed in future studies. First, the US assessment of nerves was performed in an ideal setting on linear portions of nerves with the superficial layer of skin being removed. The nerves were scanned in the medial bicipital groove, running superficially and not approaching joints or piercing the muscles, therefore findings are not directly transferable to other anatomical regions. Second, the sample size was too small to have sufficient statistical power; however, the nature of this study was explorative, aiming to compare the feasibility of those two methods in 3D reconstructions. Third, performing MRI scans on lower-field devices such as 3-T MRI which are most commonly employed and approved for clinical use, could reveal different findings. However, high-field 7-T MRI systems have established their importance in visualizing interneural architecture, offering a significantly improved signal-to-noise ratio, and in combination with advanced coils, even fascicles can be precisely differentiated and thus evaluated [37].

In conclusion, 3D HRUS can provide valuable supplementary data in nerve sonography, with fascicle volume measurements comparable to MRM in intraoperative settings. However, its limitations in visualizing interfascicular connections and small fascicles should be carefully considered.

## Data Availability

The datasets used and/or analyzed during the current study are available from the corresponding author on reasonable request.

## Abbreviations

2D: Two-dimensional
3D: Three-dimensional
CSA: Cross-sectional area
FVR: Fascicle volume ratio
HRUS: High-resolution ultrasound
MRI: Magnetic resonance imaging
MRM: Magnetic resonance microscopy
STIR: Short-tau inversion-recovery
US: Ultrasonography/Ultrasound
